# Leiomyosarcoma with widespread metastases in a capybara

**DOI:** 10.1177/10406387221106252

**Published:** 2022-06-27

**Authors:** Emma A. Borkowski, Justine Shotton, Joan A. Smyth

**Affiliations:** Department of Pathology and Infectious Disease, University of Surrey, School of Veterinary Medicine, Guildford, Surrey, United Kingdom; Marwell Wildlife, Colden Common, Hampshire, United Kingdom; Department of Pathobiology and Veterinary Science, University of Connecticut, Storrs, CT, USA

**Keywords:** capybaras, histiocytoma, leiomyosarcoma, neoplasia, uterus

## Abstract

A 10.5-y-old intact female capybara (*Hydrochoerus hydrochaeris*) with a history of chronic weight loss was euthanized following discovery by palpation of a large intra-abdominal mass. Postmortem examination revealed a large, firm, tan mass expanding the uterine body and extensively adhered to the jejunum and abdominal wall. Numerous pinpoint to 3-cm diameter, tan-to-red, raised masses were present throughout the parietal peritoneum, liver, lungs, and intestinal serosa. Histologic examination of the uterine mass revealed well-differentiated smooth muscle intermixed with abundant collagen, interspersed with a highly anaplastic spindle cell population extending to the serosa; the masses in the lung, liver, and peritoneum were histologically very similar to the anaplastic uterine spindle cells. Immunohistochemical staining of the uterus and lung confirmed smooth muscle origin of the anaplastic cells. To our knowledge, leiomyosarcoma has not been reported previously in a capybara, and the widespread metastases in this case represent an unusually aggressive presentation of this rare malignancy. The animal also had an incidental dermal histiocytoma, a tumor that has also not been reported previously in this species, to our knowledge.

Capybaras (*Hydrochoerus hydrochaeris*) are the largest extant species of rodent. They are indigenous to wetlands and forests of the Amazon basin and are commonly held in zoologic collections.^1,9^ Numerous parasitic and bacterial diseases have been described in free-ranging capybaras, including brucellosis,^14^ paramphistomosis,^4^ and trypanosomosis.^6^ In addition, wild capybaras have been incriminated in transmission of disease agents such as *Leishmania* spp., *Sarcocystis* spp., *Neospora* spp., *Encephalitozoon* spp., and orthopoxvirus to domestic animals and/or humans.^1,19^ In contrast, there are few reports of disease in captive capybara.^9^ Fatal placental subinvolution was reported in an adult female,^10^ and neoplasia has only been reported twice: an intramuscular fibrosarcoma in an aged male^17^ and a cutaneous squamous cell carcinoma with regional lymph node metastasis in an adult male.^9^ We describe here leiomyosarcoma in an aged capybara.

A 10.5-y-old intact female capybara, housed in a zoologic collection with 3 other female capybaras, was examined under general anesthesia because of accelerating weight loss following a period of more gradual weight loss. No males had been present in the collection during the previous 10 y, and the animal had never been pregnant. During examination, a large abdominal mass was palpated. Given the animal’s advanced age and poor prognosis, the capybara was euthanized.

At postmortem examination, the animal weighed 50.9 kg and had moderate symmetrical decreases in adipose reserves and muscle mass. The uterine body was markedly expanded by a spherical, 20-cm diameter, firm, tan mass that was extensively adhered to the abdominal wall and jejunum (Fig. 1A). Numerous pinpoint to 3-cm diameter, raised, tan-to-red plaques were distributed across the parietal peritoneum and gastrointestinal serosa (Fig. 1A). Firm, flat-surfaced, tan nodules of pinpoint to 2-cm diameter were visible from the hepatic capsular surface (Fig. 1B) and comprised ~10% of the hepatic parenchyma on cut surface. Occasional 0.2–1.0-cm diameter, firm, tan nodules were also present in all lung lobes. In addition, the entirety of the right middle lung lobe and the ventral-most margins of the right cranial and caudal lung lobes were distended by three, 15-cm diameter, air-filled bullae. A firm, 3-cm diameter, raised, flat-surfaced, alopecic, white dermal plaque was present on the right flank. The dermal plaque was solid and homogeneously tan on cut surface, with a depth of 1.5 cm.

**Figure 1.**
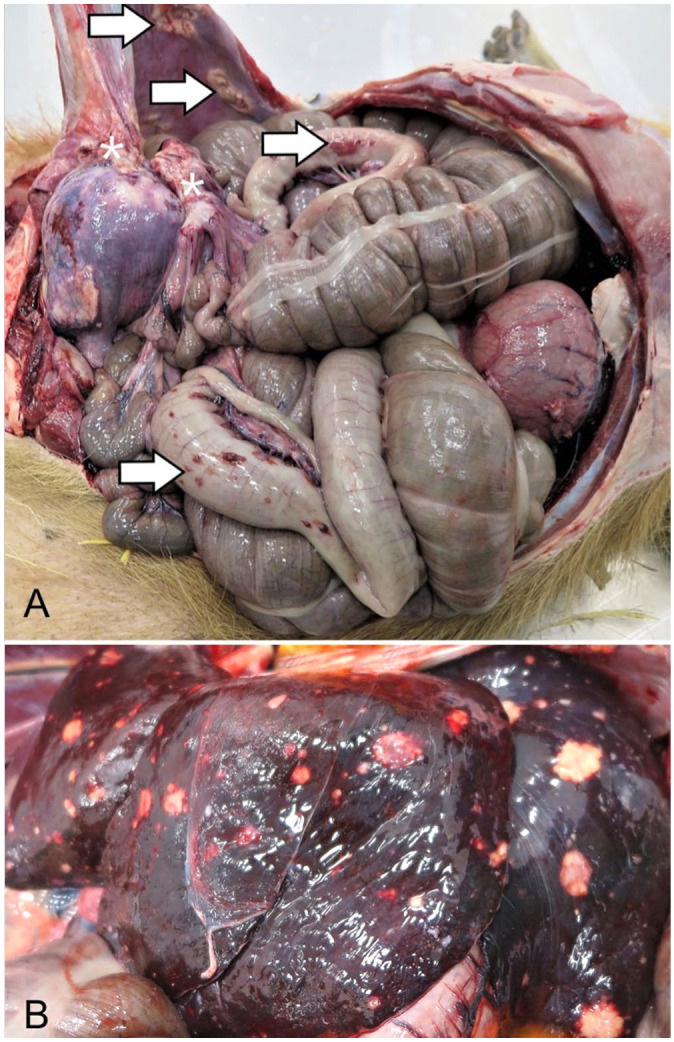
Leiomyosarcoma with widespread metastases in a 10.5-y-old intact female capybara. **A.** In the opened abdomen, there are adhesions between a large uterine body mass and the intestine and body wall (asterisks), and metastatic masses are scattered on the gastrointestinal serosa and body wall (arrows). **B.** Flat-surfaced, tan, pinpoint to 2-cm nodules are visible from the capsular surface of the liver.

Samples of all lesions were preserved in 10% neutral-buffered formalin for histologic examination. Paraffin-embedded sections of the serosal region of the uterine mass and a lung nodule were immunohistochemically stained for cytokeratins, vimentin, and smooth muscle actin by the University of Glasgow Veterinary Diagnostic Service (UG-VDS). In addition, a section of the flank mass was stained with toluidine blue at the University of Surrey, and for ionized calcium-binding adapter molecule 1 (IBA1) by UG-VDS (Table 1). All stains used had confirmed reactivity in rodent species by the manufacturer (Abcam) except cytokeratin; however, the presence of strong cytoplasmic reactivity for cytokeratins in the bronchial epithelium of the lung (internal positive control) confirmed appropriate cellular targeting of cytokeratin in the capybara.

**Table 1.** Immunohistochemical stains used to characterize uterine and dermal tumors in a 10.5-y-old intact female capybara.

Marker	Species of origin	Clonality	Dilution	Reactivity confirmed in rodents by manufacturer (Abcam)*
Cytokeratin	Mouse	MFN116	1:100	No†
Vimentin	Mouse	V9	1:5,000	Yes
SMA	Mouse	1A4	1:200	Yes
IBA1	Rabbit	Polyclonal	1:1,500	Yes

IBA1 = ionized calcium binding adapter molecule 1; SMA = smooth muscle actin.

* Rodent reactivity information derived from product monographs available at https://www.abcam.com/ (last accessed 2021 Oct 28).

† Reactivity confirmed by appropriate positive labeling of bronchial epithelium in the test section.

Microscopic examination revealed that the uterine mass consisted mainly of a well-demarcated, expansile mass of well-differentiated smooth muscle cells interspersed with broad bundles of dense collagen with low numbers of slender fibrocytes (Fig. 2A, 2B). However, highly anaplastic cells were interspersed among the well-differentiated myocytes, in progressively higher numbers toward the serosal aspect of the mass. The anaplastic cells were spindle-shaped to polyhedral with variably distinct cytoplasmic margins, highly variable amounts of eosinophilic cytoplasm, oval-to-angular nuclei with coarsely stippled chromatin, and 20 mitoses per 10 hpf (2.37 mm^2^; Fig. 2C, 2D). Mononuclear and multinuclear giant cells, as well as smaller multinucleate cells, were frequent, and anisokaryosis was marked, with nuclei enlarged up to 10-fold. The progressive increase in the anaplastic cell population was accompanied by a reduction in number and thickness of collagen bundles. The pulmonary (Fig. 2E) and hepatic (Fig. 2F) masses consisted of poorly circumscribed aggregates of dense collagen fibers interspersed with anaplastic cells similar to those described in the uterus. The anaplastic cells in both the uterus and lung displayed strong, uniform cytoplasmic reactivity for both vimentin (Fig. 3A, 3C) and smooth muscle actin (Fig. 3B, 3D), and no reactivity to cytokeratin immunolabeling. These findings confirmed leiomyosarcoma with metastasis.

**Figure 2.**
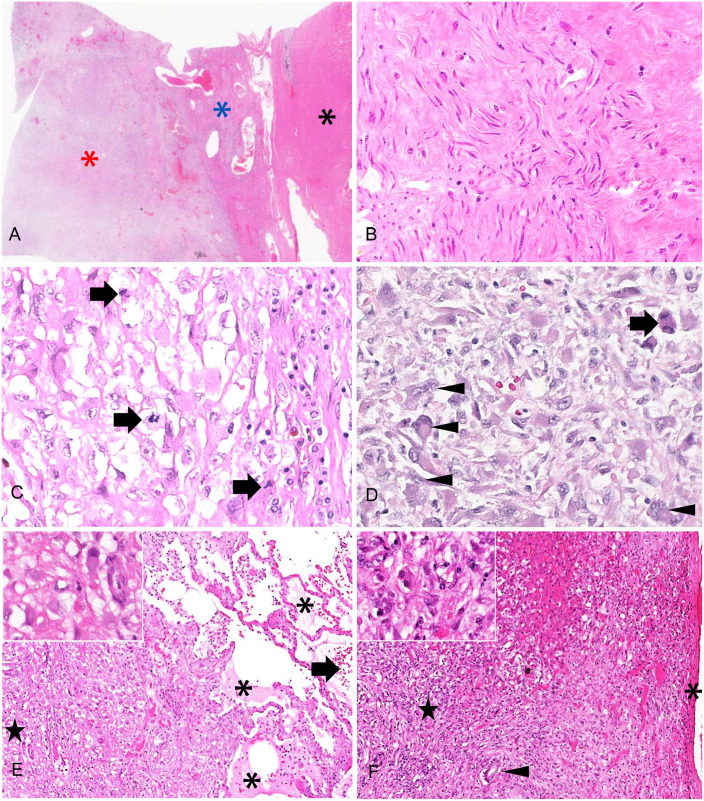
Uterus, lung, and liver of a 10.5-y-old intact female capybara. **A.** Subgross microscopic appearance of the uterine mass showing progression from a strongly eosinophilic region of low cellularity (black asterisk) through a vascular transition zone (blue asterisk) to a highly cellular population at the serosal surface (red asterisk). H&E. **B.** Higher magnification of the region indicated by the black asterisk in Fig. 2A. The myometrium is expanded by a mass of irregularly oriented, slender, well-differentiated smooth muscle cells separated by abundant collagenous matrix. H&E. **C.** Higher magnification of the region indicated by the blue asterisk in Fig. 2A. Anaplastic cells effacing the myometrium. Several mitotic figures are present (arrows), and there are collagen fibers at the right of the image. H&E. **D.** Higher magnification of the region indicated by the red asterisk in Fig. 2A. Anaplastic cells are interspersed with scant thin collagen fibers at the serosal surface of the uterus. A mitotic figure (arrow), and large multinucleate cells (arrowheads) are present. H&E. **E.** A poorly circumscribed infiltrative mass of anaplastic cells admixed with collagen, effacing alveoli in lung. There is proteinaceous fluid (asterisks) and hemorrhage (arrow) in the adjacent unaffected alveoli. Inset: higher magnification of anaplastic cells within the lung, taken from the region marked on the larger image by the black star. H&E. **F.** A poorly circumscribed, infiltrative mass of anaplastic cells admixed with collagen effacing hepatic parenchyma. A bile ductule is entrapped within the neoplasm (arrowhead); the capsule is present on the right (asterisk). Inset: higher magnification of anaplastic cells within the liver, taken from the region marked on the larger image by the black star. H&E.

**Figure 3.**
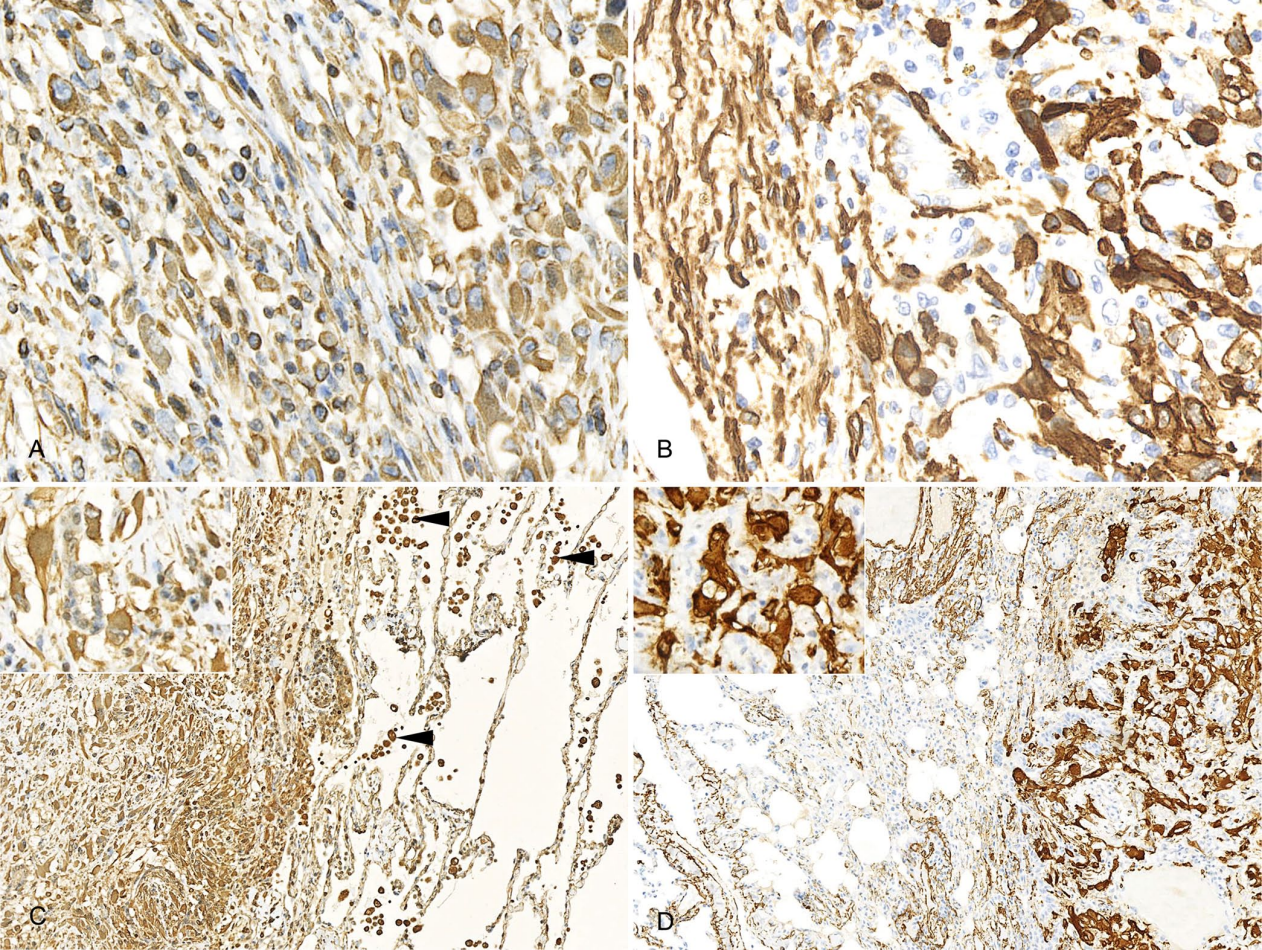
Immunocytochemically stained histologic sections of uterus and lung of a 10.5-y-old intact female capybara. **A.** Intense cytoplasmic immunolabeling of vimentin in the neoplastic cells in the uterine mass. Anaplastic cells are present throughout the image and are interspersed with slender, well-differentiated myocytes at the left of the image. This image corresponds to the transition region marked by the blue asterisk in Fig. 2A. **B.** Intense cytoplasmic immunolabeling of smooth muscle actin in the neoplastic cells in the uterine mass. A band of slender, well-differentiated myocytes is present at the left of the image. This image corresponds to the transition region marked by the blue asterisk in Fig. 2A. **C.** Intense cytoplasmic immunolabeling of vimentin in the neoplastic cells in the lung. Labeling is also present in alveolar macrophages at the right of the image (arrowheads; internal positive control). Inset: higher magnification of the neoplastic cells within the lung. **D.** Intense cytoplasmic immunolabeling of smooth muscle actin in the neoplastic cells in the lung. Inset: higher magnification of the neoplastic cells within the lung.

The dermal plaque on the right flank consisted of a well-demarcated, unencapsulated aggregate of large neoplastic round cells forming variably sized clusters separated by thick, densely arranged collagen bundles, with numerous eosinophils intermixed. The overlying epidermis was markedly thickened and formed prominent rete pegs. The neoplastic cells had variably distinct cytoplasmic margins and abundant faintly eosinophilic cytoplasm, rarely containing scant, basophilic granules that were not metachromatic with toluidine blue stain. Nuclei were round-to-oval with dispersed chromatin and prominent, single nucleoli. Rare nuclei displayed indentation; anisokaryosis was mild with occasional binucleate cells throughout the mass; and mitotic count was 28 per 10 hpf (2.37 mm^2^). All neoplastic round cells displayed moderate-to-strong reactivity to IBA1; half of the population had membranous reactivity and the remaining half had cytoplasmic reactivity, confirming histiocytic origin. The low degree of cellular atypia was considered most consistent with histiocytoma.

Based on the gross and histologic findings, 2 possible origins were considered for the leiomyosarcoma: 1) uterine origin with serosal and hematogenous metastasis, and 2) an unidentified primary site with hematogenous and serosal metastasis, including to the uterus. Uterine origin was considered most likely for several reasons. First, although the bulk of the large uterine mass had a relatively benign appearance consistent with fibroleiomyoma, there was evidence of progressively increasing anaplasia approaching the serosal surface. This could be consistent with a slow-growing fibroleiomyoma in which the neoplastic cells gradually accumulated additional mutations, allowing for the eventual appearance of a highly malignant subpopulation of neoplastic cells capable of breaching the serosa and metastasizing locally and systemically. The pattern of gradual progression from well-differentiated to anaplastic cells is also not consistent with deep invasion of a serosal metastatic focus, which would be expected to form invasive cords of anaplastic cells extending into and surrounded by the deeper, well-differentiated tissue, as was observed in the hepatic and pulmonary metastases. Such cords were not observed; moreover, the densely collagenous structure of the uterine neoplasm would have acted as a barrier to deep invasion of a serosal metastasis from another primary site.

Uterine smooth muscle tumors are uncommon in animals, although benign and/or malignant tumors have been reported in a range of species including nondomestic felids^3,11^ and suids^7^ maintained in zoologic collections, goats,^5^ dogs,^18^ and domestic cats.^15^ To date, there have been no reports of uterine tumors in capybara. Guinea pigs, the closest domestic relative of the capybara, frequently develop uterine tumors; however, most are leiomyomas. Leiomyosarcoma was identified concurrent with choriocarcinoma in one animal in a case series of 23 guinea pigs with uterine lesions, of which 13 of 37 lesions were neoplasms.^20^ Prevalence of uterine leiomyosarcoma in guinea pigs ranges from reports of 5 of 62 uterine neoplasms^2^ to 9 of 83 uterine proliferative lesions.^12^ Two uterine leiomyosarcomas were also reported in a case series of 18 reproductive neoplasms in guinea pigs of both sexes, although reproductive neoplasia comprised only 18 of 341 neoplasia cases in that study.^16^ Thus, the incidence of uterine leiomyosarcoma in the closest relative of the capybara is also low. In all species, reports of uterine leiomyosarcoma without metastases greatly outnumber cases with confirmed metastases; thus, our case is also unusual because of the widespread metastases. Reports of leiomyosarcoma have largely described differentiation suggestive of smooth muscle origin based solely on routine H&E staining, without requiring immunohistochemistry unless a second neoplastic population is present.^3,5,20^ Thus, the neoplasm described here was also unusual in its high degree of anaplasia.

The risk factors for development of malignant uterine neoplasms are incompletely understood. Notably, although captive wildlife are often administered contraceptive treatment, which has been associated with non-neoplastic genital tract lesions and some reproductive neoplasms, no association has been found between contraceptive exposure and uterine smooth muscle tumor development.^3,7^ There was no history of contraceptive administration to the female capybara in this collection. Age is well-recognized as a risk factor for uterine smooth muscle neoplasms in various species.^7,20^ Given that the typical lifespan of a capybara is 8–10 y,^9^ the advanced age of this animal was likely the primary predisposing factor.

The large pulmonary bullae and the flank histiocytoma in this capybara are also of interest, although unrelated to the uterine neoplasm and weight loss. Pulmonary bullae have not been reported in capybaras and do not appear to be a common lesion in aging guinea pigs but may be seen in guinea pigs used as a model for smoke exposure–related emphysematous changes.^8^ The bullae in our case more closely resembled the idiopathic pulmonary bullae seen relatively commonly in dogs, especially deep-chested breeds.^13^ Such bullae can occasionally rupture and cause spontaneous pneumothorax, but this complication had not occurred in our case. Histiocytoma has also not been reported in a capybara to our knowledge, and no cases were reported in a series of 341 neoplasms in aging guinea pigs^16^; hence, histiocytoma appears to be an uncommon lesion in this family of rodents.
